# Covalent Bruton tyrosine kinase inhibitors across generations: A focus on zanubrutinib

**DOI:** 10.1111/jcmm.70170

**Published:** 2025-01-31

**Authors:** Alessandro Broccoli, Marzia Del Re, Romano Danesi, Pier Luigi Zinzani

**Affiliations:** ^1^ IRCCS Azienda Ospedaliero‐Universitaria di Bologna Istituto di Ematologia “Seràgnoli” Bologna Italy; ^2^ Dipartimento di Scienze Mediche e Chirurgiche Università di Bologna Bologna Italy; ^3^ Department of Clinical and Experimental Medicine University of Pisa Pisa Italy; ^4^ Department of Oncology and Hemato‐Oncology University of Milano Milan Italy

**Keywords:** BTK inhibitors, chronic lymphocytic leukemia, follicular lymphoma, mantle cell lymphoma, marginal zone lymphoma, Waldenstrom macroglobulinemia, zanubrutinib

## Abstract

Bruton tyrosine kinase (BTK), the primary target of BTK inhibitors, is a key enzyme in the proliferation and survival pathway of neoplastic B‐cells. BTK inhibitors are approved in many hematologic malignancies: chronic lymphocytic leukaemia, mantle cell lymphoma, marginal zone lymphoma, Waldenström macroglobulinaemia and follicular lymphoma. Second‐generation BTK inhibitors display high target selectivity thus resulting in a reduction in off‐target and off‐tissue effects, better therapeutic index and improved tolerability. This paper summarizes the mechanisms of action of first and second generation BTK inhibitors and elucidates results in any disease setting, with a precise focus on zanubrutinib.

## 1 INTRODUCTION

Kinases are key regulatory components of the pathogenesis of several blood diseases. Targeting of tyrosine kinases with small molecules, given orally for an indefinite period of time, has become a well‐established treatment strategy in chronic myeloid malignancies and—more recently—also in chronic B‐cell lymphoproliferative diseases. The mechanism of kinase inhibition mainly rests on the binding of the inhibitor to the ATP‐binding site of the enzyme. However, similarities in the structure of the ATP‐binding pocket across different kinases has represented a challenge in developing selective inhibitors. This implies that each small molecule inhibitor has more than one target, although binding to each target is realized with a different affinity.^1^

Bruton tyrosine kinase (BTK) is a key component of the B‐cell receptor (BCR) signalling pathway, being essential for B‐cell development but also being implicated in proliferation and survival of malignant B‐lymphocytes. Given the central role of BTK in B‐cell diseases and its relatively restricted expression, BTK inhibition has emerged as an attractive and promising way to treat multiple relapsed lymphoproliferative syndromes, ranging from chronic lymphocytic leukaemia (CLL) to mantle cell lymphoma (MCL), Waldenström macroglobulinaemia (WM) and, more recently, marginal zone lymphoma (MZL).^2^ Results obtained with the first‐in‐class BTK inhibitor ibrutinib in pretreated B‐cell malignancies have also prompted its use in the treatment‐naïve setting, in particular with clinically meaningful results in patients harbouring adverse clinical or cytogenetic or molecular features that may configure high‐risk or chemotherapy‐unresponsive diseases.^3^

Acalabrutinib, zanubrutinib, and other covalent BTK inhibitors (like tirabrutinib and orelabrutinib) have been developed after ibrutinib, with the aim of narrowing the spectrum of inhibited kinases, thus trying to reduce the potential off‐target effects, mainly in terms of adverse events. More recently, non‐covalent molecules have been applied in cases of disease refractoriness or progression under covalent BTK inhibition.^4^

This paper reviews the pharmacological properties, the efficacy, and the safety profiles of the most widely applied—and approved—covalent BTK inhibitors (ibrutinib, acalabrutinib, and zanubrutinib), with the specific purpose of underscoring the peculiarities that make them belong to different drug ‘generations’. We will focus specifically on zanubrutinib, which is the latest BTK inhibitor to come to the clinical scenario and to the market, with approval in 5 indications so far.

## 2 PHARMACOLOGIC PROPERTIES OF COVALENT BTK INHIBITORS

### 2.1 Ibrutinib

**FIGURE 1:**
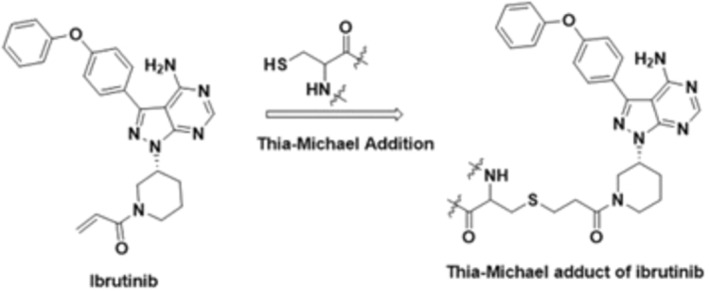
Covalent binding of ibrutinib to its primary target (from^5^ reproduced with permission).

Ibrutinib is a potent, irreversible, covalent inhibitor of BTK, which binds with the cysteine (Cys) 481 at the active site of BTK (Figure 1) via Thia‐Michael addition reaction between the thiol group of cysteine and the acrylamide group of ibrutinib. BTK is a tyrosine kinase expressed in the hepatocellular carcinoma (TEC) family of tyrosine kinases and is an essential element of the BCR signalling pathway. Genetic knockout or inactivation of BTK in mice produces predominantly a B‐cell defect with absent B1 lymphocytes, diminished B cells, and disrupted BCR signalling. Preclinical work with ibrutinib demonstrated disruption of BCR signalling and in vivo activity in lymphoma models with documented targeted inhibition of BTK. Preclinical trials demonstrated that ibrutinib promotes apoptosis of CLL cells, inhibits activation of phosphatidylinositol‐3 kinase (PI3K), ERK1, and NF‐κB by external microenvironment signals, and also prevents CLL proliferation.^6^ Ibrutinib is rapidly absorbed after oral administration, with peak plasma concentrations observed 1 to 2 h after dosing. Its exposure increases with doses up to 840 mg/day. The steady‐state area under the curve (AUC) observed in patients at 560 mg/day is 953 ± 705 ng ⋅ h/mL and in patients at 420 mg/day is 680 ± 517 ng ⋅ h/mL. A phase 1, open‐label, dose‐escalation trial demonstrated that administration of ibrutinib with food increases exposure approximately two‐fold compared with administration after overnight fasting. Further pharmacokinetic (PK) data showed 30% higher AUC values in patients ages 65 years and older at a dose of 420 mg/day.^6^ However, greater concentrations did not confer accumulation or increased toxicity; therefore, ibrutinib may be administered with or without food and dose reductions are not advised in elderly patients. Ibrutinib exposure in males and females is similar. No substantial difference in systemic exposure was found between patients with deletion of chromosome 17p (del17p) versus those who were negative for this cytogenetic abnormality.^6^

Ibrutinib has a very large apparent volume of distribution at steady state of approximately 10,000 L, which suggests that the drug easily and widely accesses the intracellular compartment. Metabolism is the main route of elimination for ibrutinib. It is converted to several metabolites, primarily by the cytochrome P450 (CYP) enzyme CYP3A4, and to a minor extent by CYP2D6. The active metabolite has inhibitory activity towards BTK approximately 15 times lower than that of ibrutinib. The range of the mean metabolite to parent ratio at steady state is 1:2.^6^

Ibrutinib plasma concentrations follow a biphasic elimination pattern, resulting in an apparent terminal half‐life of 4–6 h. It is eliminated primarily via the faeces (approximately 80%), mainly in the form of metabolites (only 1% as unchanged drug). The elimination of ibrutinib in the urine is less than 10% and in the form of metabolites.

Post‐treatment assessments of ibrutinib in CLL patients showed full inhibition of BTK in peripheral blood mononuclear cells at doses of 420 and 840 mg per day. The median level of BTK occupancy was 96%–99%, which was seen as early as 4 h after administration and maintained for 24 h at both dose levels.^6^

### 2.2 Acalabrutinib

Acalabrutinib is a highly selective, potent, second generation, covalent BTK inhibitor. After oral administration, acalabrutinib is rapidly absorbed and has a short PK half‐life; the median time to reach peak plasma concentrations (*T*_max_) is 0.9 h (range: 0.5–1.9 h). After a single oral dose (100 mg), the terminal elimination half‐life of acalabrutinib is 1–2 h. ACP‐5862, the major pharmacologically active metabolite of acalabrutinib, is approximately half as potent as acalabrutinib in terms of BTK inhibition, has a similar kinase selectivity profile, and has a half‐life of terminal elimination of approximately 7 h.^7^

The formation of ACP‐5862 is also characterized by a *T*_max_ of approximately 1 h and a mean exposure double that of acalabrutinib. Both acalabrutinib and ACP‐5862 are irreversible covalent kinase inhibitors and inhibition of signal transduction is maintained until functional levels of the target kinase are restored.^7^ Median stable BTK occupancy at baseline (≥ 95%) in peripheral blood mononuclear cells was maintained for 12 h with administration of 100 mg acalabrutinib twice daily in patients with B‐cell malignancies. ACP‐5862 is originated from acalabrutinib primarily by CYP3A‐mediated oxidation of its pyrrolidine ring. In a study on the absorption, distribution, metabolism, and excretion of acalabrutinib in humans, 95.7% of the sum of drug and metabolite were recovered primarily in faeces (83.5%) and to a minor extent in urine (12%). In human faeces, the parent acalabrutinib represented 1.2% of the excreted dose, suggesting that metabolic clearance by the liver plays a prominent role in total body clearance.^7^ The results of these analyzes indicate that metabolic clearance is the primary route of drug elimination of acalabrutinib in humans.

Acalabrutinib has a large steady‐state volume of distribution (101 L for acalabrutinib and 67 L for ACP‐5862), suggesting that the drug easily accesses the intracellular compartment. For the exposure–efficacy analysis, no clinically meaningful relationships were observed between total AUC and efficacy outcomes including best objective response and progression‐free survival (PFS) following treatment with acalabrutinib in patients with previously untreated CLL. Similarly, no relationships were observed between acalabrutinib exposure and incidences of grade ≥2, grade ≥3, or selected grade ≥2 adverse events of clinical interest following treatment with acalabrutinib in patients with B‐cell malignancies. The lack of relationship between exposure and efficacy and safety endpoints indicates that the acalabrutinib 100‐mg twice‐daily dosing regimen elicits potent and consistent therapeutic effects across the exposure range observed.^7^

Acalabrutinib has a very high selectivity and potency. Despite the short half‐life, acalabrutinib had a pronounced on‐target impact in peripheral blood B cells that was dose dependent. Complete BTK occupancy was observed 3 and 12 h after a single 100‐mg dose, indicating that a saturating concentration was achieved that resulted in near complete inhibition of a BCR‐induced functional response (i.e., CD69 expression). Both measures remained high for 24 h.^8^ Therefore, covalent modification of BTK by acalabrutinib prolonged target occupancy and PD that extend beyond the relatively short plasma half‐life in healthy volunteers.^8^

### 2.3 Zanubrutinib

Zanubrutinib is an oral, irreversible BTK inhibitor. Like ibrutinib, zanubrutinib forms an irreversible, covalent bond at Cys481 within the adenosine triphosphate‐binding pocket of BTK. Zanubrutinib is more selective than ibrutinib against off‐target kinases, including epidermal growth factor receptor (EGFR), Janus kinase 3 (JAK3), TEC and IL‐2 inducible tyrosine kinase (ITK), based on results from kinase inhibition and cell‐based assays.^9^ For example, zanubrutinib demonstrated selectivity of 88‐fold, 13.8‐fold, and 2754‐fold against TEC, HER4 and JAK3, respectively. The increased BTK selectivity is thought to result in a lower incidence and severity of off‐target toxicities linked to inhibition of the aforementioned kinases.^9^ Zanubrutinib maintains similar or improved potency for BTK inhibition compared with other molecules in the class. The IC_50_ values can vary slightly depending on the type of assay and conditions. When comparing IC_50_ values of zanubrutinib and ibrutinib directly in the same biochemical assay, the estimated IC_50_ values were 0.3 and 0.18 nM for zanubrutinib and ibrutinib, respectively.^9^

Zanubrutinib was rapidly eliminated, with a mean terminal elimination half‐life (t_½_) of approximately 2 to 4 h. There was a dose‐proportional increase in maximum concentration (C_max_) and AUC at doses from 40 to 320 mg. Following multiple‐dose administrations of zanubrutinib, limited systemic accumulation was observed at all doses, which is consistent with its t_½_. Based on noncompartmental PK analysis, the geometric mean (%CV) zanubrutinib steady‐state daily AUC is 2295 (37%) ng ⋅ h/mL following 160‐mg twice daily and 2180 (41%) ng ⋅ h/mL following 320‐mg once‐daily dosing. The geometric mean (%CV) steady state *C*_max_ is 314 (46%) ng/mL following 160 mg twice‐daily and 543 (51%) ng/mL following 320 mg once‐daily dosing. Consistent with the preclinical prediction, zanubrutinib achieved higher bioavailability than ibrutinib, with estimated oral bioavailability of 15% based on the physiologically based PK (PBPK) model relative to 3.9% (fasting state) for ibrutinib.^9^ The sustained plasma exposures achieved by zanubrutinib translate into a durable BTK inhibition in target tissues. At the 320 mg total daily dose, zanubrutinib achieved median steady‐state BTK occupancy of 100% in peripheral blood mononuclear cells for more than 24 h. In addition, BTK inhibition was assessed in study AU‐003 in pre‐ and on‐treatment lymph node biopsies, demonstrating a median steady‐state (trough) BTK occupancy of 94% and 100% following the 320 mg once‐daily and 160 mg twice‐daily regimens, respectively. These results indicate efficient and sustained inactivation of BTK in target tissues throughout the recommended dosing interval and are consistent with high rates of objective responses reported in zanubrutinib‐treated patients with B‐cell malignancies.^9^

## 3 CLINICAL APPLICATIONS IN CHRONIC LYMPHOPROLIFERATIVE DISEASES

### 3.1 Chronic lymphocytic leukaemia

CLL and small lymphocytic lymphoma (SLL) are the disease in which BTK inhibitors have been more extensively employed, both in the relapsed and refractory setting and in treatment‐naïve patients, across the entire spectrum of cytogenetic and molecular risk features.

The pivotal experience with single‐agent ibrutinib in a cohort that included relapsed/refractory patients (median four prior therapies) and frontline‐treated cases (patients aged 65 years or more), now reaching its longest follow‐up of 85 months, demonstrates long‐term responses—albeit predominantly partial responses (PR)—with a median duration of response (DoR) not reached in patients treated frontline, and 57 months in patients treated for relapsed/refractory disease. PFS at 7 years was 83% and 34% for frontline and pretreated patients, respectively; a trend towards longer PFS was observed in patients receiving less lines of treatment (one or two) rather than more than four. Patients with adverse cytogenetic features, namely del17p and del11q, reached a median PFS at 26 and 51 months, respectively.^10^ In an independent study focusing on frontline‐treated patients with *TP53* disruptions (del17p in 92% of the cases and *TP53* mutations in 8%), the 6‐year PFS was 61%, with an overall survival (OS) of 79%.^11^ Phase 3 randomized trials have explored the role of ibrutinib in the relapsed setting, compared to ofatumumab (RESONATE), and in the frontline context, compared to single‐agent chlorambucil (RESONATE‐2).^12^, ^13^ The RESONATE study involved 195 patients in the ibrutinib arm, more than half of them having received at least three previous lines of therapy and presenting with del17p, immunoglobulin heavy chain variable region gene (*IGHV*) unmutated status, and *TP53* mutations in 32%, 73% and 51% of the cases, respectively. Apart from demonstrating a clear superiority of ibrutinib over ofatumumab and a median PFS for the entire ibrutinib‐treated population of 44 months, this study showed no significant PFS difference for patients with or without del17p, *IGHV* mutated or unmutated status and presence or absence of complex karyotype. However, the presence of *TP53* mutations still trended over worse PFS, although patients with either del17p or *TP53* mutations fared similarly to those with both lesions.^14^ Patients in the RESONATE‐2 study were all 65 years or older, with a cumulative illness rating scale (CIRS) score higher than 6 in one‐third of the cases and a creatinine clearance <60 mL/min in nearly half of the cases in each arm, thus not qualifying for chemoimmunotherapy. Importantly, this study did not include patients with del17p, although 9% of *TP53*‐mutated patients and 57% of *IgHV* unmutated cases were present in the ibrutinib arm. A statistically significant difference was observed for ibrutinib over chlorambucil in both PFS and OS, with both median PFS and OS for ibrutinib frontline‐treated patients not reached with up to 8 years of follow‐up. The *IGHV* mutational status did not affect PFS of patients receiving ibrutinib in the long run.^15^

Similar to ibrutinib, acalabrutinib was also initially explored in a phase 2 trial in 134 relapsed and refractory CLL/SLL patients, regardless of age, who had received a median of two previous treatments and had del17p, unmutated *IGHV* status, and complex karyotype in 23%, 73% and 35% of the cases, respectively. Ninety‐four percent of the patients responded, with a complete response (CR) in 4% of the cases and a PR + PR with lymphocytosis in 90% of the cases. Patients with adverse cytogenetic and molecular alterations also responded in more than 90% of the cases. Although the median PFS for the entire population was not reached at a median follow‐up of 41 months, it was 36 and 33 months for patients with del17p and complex karyotype, respectively.^16^ In the relapsed and refractory setting, acalabrutinib proved its superiority over chemotherapy with bendamustine and rituximab or idelalisib plus rituximab therapy (chosen at investigator's discretion) in the phase 3 ASCEND trial,^17^ and proved to be non‐inferior to ibrutinib, in terms of PFS, in a head‐to‐head comparison.^18^ As far as frontline treatment is concerned, the ELEVATE‐TN trial randomized 535 patients to either receive acalabrutinib (A), acalabrutinib + obinutuzumab (AO), or chlorambucil + obinutuzumab. Patients were 65 years or older and had comorbidities precluding chemoimmunotherapy; del17p and/or *TP53* mutations were documented in 14% of the patients and an unmutated *IGHV* gene was found in 63% of cases. Both the acalabrutinib‐containing arms demonstrated improved PFS over chlorambucil + obinutuzumab. Statistically significant enhanced PFS was seen in the AO arm over A only at 48 months (87% vs. 78%, *p* < 0.0296). According to cytogenetic and molecular features, the 48‐month PFS rates in the AO and A arms were 75% and 76%, respectively, for patients with del17p and/or mutated *TP53*, and 86% and 77% for patients with unmutated *IGHV*.^19^

Data on single‐agent zanubrutinib in treatment‐naïve and relapsed or refractory patients with CLL and SLL come from a pooled analysis of 3 phase 1/2 trials,^20^, ^21^, ^22^, ^23^ two of them conducted specifically in a Chinese population.^22^, ^23^ In two of these trials, zanubrutinib was given at the dose of either 160 mg twice daily or 320 mg/day. Similar plasma exposure and BTK inhibition were obtained with any of the administration schedules, and differences in trough concentrations as well as maximum plasma concentrations between the two schedules were deemed unlikely to have a meaningful impact on efficacy and safety. In total, 211 patients have been included, 19 being treatment‐naïve and 192 already treated. Comparisons were made after creating a weighted sample in which covariates (age, sex, performance status, disease stage, presence of bulky masses, *IGHV* and *TP53* mutational status, peripheral blood counts) were balanced between the two groups. After weighting, the overall response rate (ORR) was 100% in the treatment‐naïve group and 91% in the relapsed/refractory group (with a CR rate of 21% vs. 9%, respectively). Moreover, patients treated with one previous line only had a significantly higher ORR than those receiving more than one line of treatment before zanubrutinib (98% vs. 91%, respectively). The PFS rate at 3 years was 78% for the entire population: 83% for treatment‐naïve and 73% for pretreated patients. Longer PFS and OS rates were documented for patients having received only one previous treatment line compared to those who were more heavily pretreated; however, no significant differences were observed between patients who had received only one prior therapy and treatment‐naïve ones. Finally, PFS and OS functions were comparable among patients with or without *TP53* disruptions.^20^ ALPINE was a randomized, phase 3 trial that compared ibrutinib and zanubrutinib in a head‐to‐head fashion in patients with relapsed and refractory CLL/SLL. Patients had received a median of one prior treatment, including BCL2 inhibitors and PI3K or spleen tyrosine kinase inhibitors (although in a limited proportion of cases). At a median follow‐up of nearly 30 months, zanubrutinib proved to be superior to ibrutinib as far as PFS was concerned, with a 2‐year PFS rate of 78% versus 66%. A consistent PFS benefit was observed in zanubrutinib‐treated patients bearing high‐risk features, namely del17p, *TP53* mutations, or both (73% vs. 55% at 2 years, respectively). Likewise, the proportion of patients with an objective response was higher in the zanubrutinib group than in the ibrutinib group (ORR of 91% vs. 83%, respectively).^24^ SEQUOIA, in contrast, evaluated the role of zanubrutinib in the frontline setting, in comparison to bendamustine and rituximab chemoimmunotherapy, in a cohort of 590 patients aged 65 years or older, or 18 years or older with comorbidities, excluding cases with del17p. At a median follow‐up of 26 months, median PFS was not reached in either group, although displaying a superiority for zanubrutinib over chemoimmunotherapy (2‐year PFS of 86% vs. 70%, respectively). A separate cohort within the study was specifically composed of patients with del17p; they all received zanubrutinib as frontline treatment and responded in 90% of the cases, with a CR in 6% of the cases. PFS and OS for these patients were 89% and 90% at 2 years, respectively.^25^

### 3.2 Mantle cell lymphoma

The first experience with ibrutinib in relapsed MCL patients was published in 2013: 111 patients received ibrutinib at the daily oral dose of 560 mg until progression or unacceptable toxicity. Patients were stratified as having received prior bortezomib or not, and in general they were more highly pretreated (55% of patients received at least three previous treatments) with intensive regimens, autologous transplantation, and lenalidomide as earlier salvage. Nearly half of patients had refractory disease and up to three quarters displayed advanced stage lymphoma. Ibrutinib produced a response in less than 2 months; ORR was not different in patients with or without prior treatment with bortezomib (67% vs. 68%, respectively), and showed a tendency to increase over time, albeit with the prevalence of partial over CR. The median DoR observed in the study population was 17.5 months and represented at that time the longest reported for a single agent in relapsed and refractory MCL. The median PFS was 13 months in the overall population, with better trends for patients previously exposed to bortezomib, with lower tumour bulks, less pretreatment, and chemosensitive disease at ibrutinib inception.^26^, ^27^

Results of single‐agent acalabrutinib in patients with pretreated MCL were published in 2018. None of the patients in the trial had received prior treatment with BTK inhibitors or BCR inhibitors in general. Patients receiving acalabrutinib showed similar characteristics to those enrolled in the ibrutinib trial in respect to age, tumour bulk, and disease stage, although they were globally less pretreated, had a lower simplified MCL international prognostic index (MIPI), and were refractory to their last therapy in 24% of the cases (vs. 45% in the ibrutinib‐treated population). Eighty‐one percent of patients responded to acalabrutinib, and a CR was achieved in 40% of them, at a median time of 1.9 and 3.4 months to overall and CR, respectively. Importantly, 94% of patients obtained a reduction in lymphadenopathy, with objective responses in 78% of those who had bulky tumours. Moreover, responses were consistent across patients with refractory disease, blastoid or pleomorphic variant, and higher Ki‐67 index (≥50%). Median DoR was 26 months, with an estimated DoR at 2 years of 52.4%; 49% of patients were progression free at 2 years.^28^, ^29^

The extended experience with zanubrutinib at the dose of 160 mg twice or 320 mg/day is summarized in a paper published in 2023^30^ pooling data of 112 patients from two clinical trials.^31^, ^32^ Stratification was according to the number of previous treatment lines they received (one, 37% of patients, vs. at least two, 63% of patients). Less pretreated patients showed a higher MIPI score, but were characterized by lower incidence of bulky disease, extranodal dissemination, and blastoid histology. Comparison between early‐ and late‐treated patients was made after propensity score weighting in order to balance covariates (age, sex, bone marrow involvement, performance status, stage, blastoid variant, MIPI, bulky or extranodal disease) to mimic randomization. After weighting, ORR appeared slightly higher in patients who received zanubrutinib as second‐line therapy compared to those who were treated later (89% vs. 86%, respectively), despite DoR remaining similar between the two groups (25 months). PFS was also similar in the two groups, although with a tendency towards better median results in less pretreated patients (28 vs. 22 months). OS, in contrast, was superior in second‐line‐treated patients (82% vs. 67% at 36 months).^30^

### 3.3 Waldenström macroglobulinaemia

Ibrutinib has been initially studied in patients with relapsed and refractory WM in need of treatment at the dose of 420 mg per day until progression or unacceptable toxicity.^33^ In 63 patients with symptomatic disease, ibrutinib was able to reduce the mean immunoglobulin M level from 3520 to 880 mg/dL, along with an increase in median haemoglobin levels from 10.5 to 13.8 g/dL and a reduction in marrow infiltration from 60% to 25%. Patients responded in 91% of the cases, with a major response (MR)—that includes the amount of CR, very good partial responses (VGPR), and PR—in 79% of the cases. Importantly, patients with the L265P mutation of *MYD88* and wild‐type (WT) for *CXCR4* achieved the highest response rates (ORR of 100% and 97% of MR). They were followed by patients with *MYD88* ^L265P^ and those with the warts, hypogammaglobulinemia, infections, and myelokathexis (WHIM) syndrome *CXCR4* (*CXCR4* ^WHIM^) mutation (ORR 86% and MR 68%), and by those with *MYD88* ^WT^ and *CXCR4* ^WT^ (ORR 50%, with no MR). The 5‐year PFS for all patients was 54%: patients with *MYD88* ^L265P^ and *CXCR4* ^WT^ had a 5‐year PFS of 70%, while it declined to 38% in patients with *MYD88* ^L265P^ and *CXCR4* ^WHIM^. Patients with both WT *MYD88* and *CXCR4* progressed early (median PFS <1 year).^34^ Following these data, ibrutinib has been applied as frontline monotherapy in 30 WM symptomatic patients, all carrying the *MYD88* ^L265P^ mutation and with *CXCR4* mutations in 47% of the cases. Statistically significant improvements in serum immunoglobulin M levels (4370 to 1513 mg/dL), haemoglobin concentration (10.3 to 13.9 g/dL), and bone marrow infiltration (65% to 20%) have been observed after ibrutinib treatment, with ORR and MR rates of 100% and 83%, respectively, for the whole population. Patients with WT *CXCR4* had deeper and more rapid responses to treatment than those carrying mutations.^35^

Acalabrutinib in WM patients who received previous treatment or who were considered ineligible for frontline chemoimmunotherapy was tested in a phase 2 multicenter European trial involving 106 patients. Among 106 treated patients, an ORR of 93% was obtained in both treatment‐naïve and relapsed or refractory patients, with an MR of 80% in each subgroup. Importantly, acalabrutinib produced an MR in 64% of patients with *MYD88* ^WT^ status, thus seeming much more potent than ibrutinib in this cohort of patients. The median time to best response was 4.6 months, with a median decline in IgM concentration of 2126 mg/dL and a median increase in haemoglobin concentration of 1.2 g/dL. Median DoR was not reached in either the treatment‐naïve or relapsed or refractory cohort, with a PFS at 2 years of 82% and 90%, respectively.^36^

Zanubrutinib was first applied in WM at the dose of 160 mg twice daily in 77 patients with symptomatic disease: 53 previously treated and 24 never treated. Responses were observed in both populations, with best VGPR rates of 33% and 49% in treatment‐naïve and pretreated patients, respectively, and PR rates of 54% and 29% in the two groups, respectively. Incidence of VGPR increased over time, being 21% at 6 months, 33% at 12 months, and 44% at 2 years. As expected, patients with *MYD88* ^L265P^ and *CXCR4* ^WT^ displayed the highest VGPR rate of 59%, with a PR rate of 28%; patients with *MYD88* ^L265P^ and *CXCR4* ^WHIM^ achieved a VGPR in 27% of the cases and a PR in 64% of the cases; *MYD88* ^WT^ patients could achieve a CR and a VGPR in 13% of the cases, respectively, while a PR was documented in 38% of the cases. Estimated 24‐months PFS was 92% for treatment‐naïve patients and 76% for relapsed and refractory patients, with OS estimates at the same timepoints of 100% and 92%, for each group, respectively.^37^ A head‐to‐head comparison of zanubrutinib and ibrutinib in symptomatic WM was performed in the phase 3 randomized ASPEN study, which included 164 relapsed and refractory patients and 37 treatment‐naïve cases. Two hundred one patients with *MYD88* ^L265P^ mutation were randomized, 102 in the zanubrutinib arm and 99 in the ibrutinib arm. Patients with *MYD88* ^WT^ constituted a separate cohort (cohort 2) and were treated with zanubrutinib only.^38^ At the latest available study report, which refers to a median follow‐up of 44 months, none of the patients in the randomized part of the study achieved a CR, irrespective of the treatment status (naïve vs. relapsed/refractory) or the drug received. The VGPR rate was 36% with zanubrutinib and 25% with ibrutinib; among patients with mutated *CXCR4*, VGPR rate was 21% with zanubrutinib and 10% with ibrutinib. The proportion of VGPR increased in both arms, being 19% and 7% for zanubrutinib and ibrutinib, respectively, at 6 months; 25% and 12% at 1 year; 30% and 19% at 2 years; and 36% and 25% at 5 years. Among the 26 patients in cohort 2 treated with zanubrutinib, one patient (4%) achieved a CR, while the VGPR and PR rates were 27% and 35%, respectively. Time to MR was similar between ibrutinib and zanubrutinib (2.83 vs. 2.79 months) in patients with *CXCR4* ^WT^ and was 2.96 months in patients in cohort 2. On the contrary, as expected, time to MR was longer in *CXCR4* mutated cases: 6.64 months for ibrutinib and 3.37 months for zanubrutinib. PFS rates at 42 months were 78% and 70% for the zanubrutinib and ibrutinib arm, respectively, with no statistically significant difference. Patients in cohort 2 had a PFS of 54% at 42 months.^39^

### 3.4 Marginal zone lymphoma

According to a phase 2 single‐arm trial, BTK inhibition with ibrutinib in relapsed MZL was effective in pretreated patients, mostly if previously exposed to rituximab rather than rituximab + chemotherapy, with an ORR of 81% versus 51%. Responses were mainly partial, with CR observed in 10% of the overall population, but were consistent across all disease subtypes, with patients affected by extranodal and splenic subtypes achieving better responses than those affected by the nodal subtype (ORR of 63% and 62% vs. 47%, respectively). PFS at 33 months was 32% and 30% for patients treated with rituximab and rituximab + chemotherapy, respectively, with a median PFS reached at 30.4 and 13.8 months, respectively. Median DoR was 27.6 months, with 48% of patients remaining in remission at 33 months.^40^

Acalabrutinib produced results similar to ibrutinib in an analogous population of relapsed patients, albeit slightly less pretreated. Likewise, responses were seen in any subtype, although extranodal disease was much more prone to response than the nodal and splenic counterparts (65% vs. 42% and 46%, respectively). Nearly 76% of patients maintained their response at 12 months: PR prevailed in patients with extranodal disease (53% vs. 25% and 36% for nodal and splenic subtypes), and 48% of patients overall achieved a disease stability. PFS at 12 months was 67%, which approximates the PFS rate observed with ibrutinib at the same timepoint.^41^

In the MAGNOLIA study, zanubrutinib demonstrated a higher proportion of responses than both ibrutinib and acalabrutinib in 66 evaluable patients who had received a median number of two previous therapies and whose disease was refractory to the immediately previous anticancer treatment in 32% of the cases. The ORR was 68%, and significant ORR were seen across all histologic subtypes: 64% in extranodal MZL, including a CR in 40% of the cases, 76% in nodal MZL, with a CR in 20% of the treated patients, and 67% in splenic lymphoma, with a CR in 8% of the cases. Response duration at 24 months was 73% for the overall population: 78% and 75% of patients with nodal and extranodal disease, respectively, maintained the response at 2 years, while response duration was not reached in the splenic MZL subgroup. A large proportion of patients (77%) who obtained a CR were progression‐free at 24 months, compared with 41% of those who did not achieve a CR, yielding PFS rates of 87% and 65%, respectively.^42^

### 3.5 Follicular lymphoma

Historically, ibrutinib has demonstrated modest activity as a single agent in follicular lymphoma (FL) patients experiencing disease relapse. In 40 patients with relapsed (65%) or refractory (35%) disease and heavily pretreated, with a median number of three previous therapies, it produced an objective response in 38% of the patients, with a CR in only 13% of the cases. At 2 years, only 20% of treated patients proved to be free of disease progression. Importantly, however, it seemed to work better in patients whose disease was sensitive to rituximab rather than in those showing rituximab refractoriness (ORR of 53% vs. 17%).^43^ Similarly, results were disappointing in a cohort of 110 advanced‐stage, rituximab‐refractory, highly pretreated patients, where ibrutinib yielded an ORR of 21% and a CR rate of 11%, with a median PFS of only 4.6 months.^44^ The role of ibrutinib was also irrelevant when added to salvage chemotherapy (bendamustine and rituximab or cyclophosphamide, vincristine, doxorubicin, prednisone, and rituximab) as shown in the randomized, phase 3, placebo‐controlled SELENE study with 347 FL patients.^45^ Ibrutinib has also been combined with lenalidomide and rituximab for the frontline therapy of patients with FL (38) and MZL (10) in a phase 1 study.^46^ In this context, the 2‐year and 5‐year PFS were 79% and 60%, respectively, albeit not dissimilar from the updated results of the RELEVANCE trial for both the rituximab‐lenalidomide and rituximab‐chemotherapy arms.^47^

No published data with single‐agent acalabrutinib in FL exists thus far, given the poor results obtained with ibrutinib in the relapsed/refractory setting. A phase 2 trial aimed at demonstrating the efficacy of acalabrutinib combined with rituximab and lenalidomide in the frontline treatment of FL patients is now recruiting.

Zanubrutinib also demonstrated modest activity in relapsed and refractory FL patients as single‐agent, with an ORR of 36% and a CR rate of 18%, and thus was not considered worthy of further development in this context as a single agent.^48^ However, when combined with obinutuzumab in a cohort of 36 relapsed/refractory FL patients, it could produce an ORR of 72%, including a CR rate of 39%.^49^ Given these premises, the randomized phase 2 ROSEWOOD study explored the efficacy of the zanubrutinib‐obinutuzumab (ZO) combination versus obinutuzumab monotherapy in 217 patients with relapsed or refractory FL after two or more previous lines. The combination treatment showed superior ORR to single‐agent obinutuzumab (69% vs. 46%), with a CR rate in each arm of 39% and 19%, respectively. A statistically significant benefit in PFS was also demonstrated for ZO, as the median PFS was 28 months versus 10 months for single‐agent obinutuzumab.^50^ These data favour the ZO combination as a novel chemotherapy‐free approach to pretreated FL patients, to be tested against the rituximab‐lenalidomide association currently applied in this context.

## 4 THE EVOLUTION OF THE SAFETY PROFILE OF BTK INHIBITORS ACROSS GENERATIONS

Apart from disease progression, toxicity represents the main cause of BTK inhibitor discontinuation (transiently or permanently), at least in the initial phases of treatment,^51^ as well as the leading reason for dosage reductions. The wide application of these drugs in several clinical contexts has allowed for the enucleation of some peculiar toxicities, shared by all the covalent BTK inhibitors and thus representing ‘class‐related’ toxic effects Haemorrhage (ranging from the tendency to bruise to muco‐cutaneous bleeding to deeper and potentially life‐threatening events), infections, atrial fibrillation, hypertension, and ventricular arrhythmias are the most relevant treatment‐emergent adverse events ‘of interest’, as they necessitate careful patient monitoring while on therapy as well as a thorough pretreatment evaluation of candidates for treatment.

The improved toxicity profile of later‐generation BTK inhibitors may be attributed to a much more specific inhibition of BTK rather than other kinases, which are indeed inhibited by ibrutinib to a certain extent. Available data support the action of ibrutinib on C‐terminal Src kinase, on the PI3K/AKT axis, and on ion currents of the cardiac potential as a mechanism through which arrhythmias could be induced. The same has not been demonstrated for acalabrutinib.^52^ Likewise, inhibition of the PI3K pathway may be responsible, at least in part, for hypertension, although alternative mechanisms have been proposed, including a down‐regulation of nitric‐oxide formation, an increase in calcium channels in arteries, and ultimately vasoconstriction.^53^ Lastly, combined BTK and TEC inhibition reduces platelet activation and degranulation but also adhesion to fibrinogen.^54^ Taken together, these observations can explain why more recent and more selective BTK inhibitors can display a more favourable safety profile, albeit the risk of such peculiar adverse events is not eliminated. Moreover, it should be noted that the increased risk of adverse events (e.g., atrial fibrillation) by BTK inhibitors is counterbalanced by risk factors normally encountered in the general population and more specifically in CLL patients (older age, male sex, baseline platelet dysfunctions observed in CLL itself).^53^ In other words, it is hard to establish whether BTK inhibition stands as an independent determinant or rather a contributing cause of the aforementioned adverse events.^55^

Safety results emerging from long‐term follow‐up of RESONATE and RESONATE‐2 in CLL patients treated with ibrutinib indicate that the incidence of grade ≥3 adverse events, as well as the rate of drug discontinuation due to an adverse event, is higher in the first year of treatment and shows a tendency to decrease in the following years. Nevertheless, the incidence of adverse events of interest remained low throughout the entire on‐therapy period, and most of the patients who developed atrial fibrillation or hypertension, for instance, displayed risk factors for either condition.^14^, ^15^ The same can be stated for acalabrutinib in CLL pretreated patients.^16^ However, in a head‐to‐head comparison within the ELEVATE‐RR trial, acalabrutinib showed a better toxicity profile than ibrutinib: most of the adverse events of any grade—with atrial fibrillation and hypertension being the most relevant—occurred less frequently with acalabrutinib, whereas headache and cough appeared to be more frequent than on ibrutinib.^18^ Bleeding events were less frequent with acalabrutinib, despite the rates of major haemorrhage being comparable between arms. The incidence of grade ≥3 infections was also comparable. Importantly, treatment discontinuation occurred less frequently in the acalabrutinib versus the ibrutinib arm (15% vs. 21%, respectively), although adverse events leading to dose interruption or dose reduction were similar in both arms.^18^ In the ALPINE trial, zanubrutinib‐treated patients were characterized by a lower incidence of treatment discontinuation than ibrutinib‐treated ones. More specifically, a lower incidence of cardiac adverse events was reported in the zanubrutinib arm versus the ibrutinib arm (21% vs. 29%, respectively), and cardiac events that lead to treatment discontinuation were occasional in the zanubrutinib arm (0.3% of patients), although occurring in 4% of the cases in those who received ibrutinib. Deaths due to cardiac causes were only reported in the ibrutinib‐treated group. The incidence of atrial fibrillation or flutter of any grade was 5% versus 13% for the zanubrutinib and the ibrutinib arms, respectively. The rate of infections of any grade, of grade ≥3, and opportunistic infections was comparable between treatments. Likewise, hemorrhagic events, including major haemorrhage, and hypertension, including grade ≥3 hypertension, were reported with similar incidence in both groups. On the other hand, neutropenia was slightly more frequent in patients receiving zanubrutinib (29%) than ibrutinib (24%), although it did not translate into a higher occurrence of febrile neutropenia of any grade.^24^ Findings of the ALPINE trial were consistent with safety results of the ASPEN trial in WM: except for neutropenia, the incidence of adverse events of particular interest was lower with zanubrutinib than with ibrutinib. More specifically, atrial fibrillation or flutter, hypertension and diarrhoea were all significantly rarer with zanubrutinib at any time during treatment, as well as bleeding events. Infectious complications occurred similarly between treatment arms within the first 12 months of treatment, although their incidence declined beyond the first year on‐therapy in zanubrutinib‐treated patients.^39^

In an ongoing phase 2 trial, patients affected by B‐cell malignancies (including CLL, SLL, MCL, WM, and MZL) who were intolerant to ibrutinib, acalabrutinib, or both have been treated with zanubrutinib, postulating that a drug shift would improve the tolerance to treatment. Importantly, most of the events occurring with ibrutinib (70%) or acalabrutinib (83%) did not recur with zanubrutinib, whereas in the case of re‐occurrence of an adverse event, it was milder or equally severe with zanubrutinib than with ibrutinib or acalabrutinib. Overall, patients on zanubrutinib experienced treatment‐related adverse events in 96% of the cases, although they were serious in only 12% and with no fatalities.^56^

## 5 SUMMARIZING REMARKS

The class of BTK inhibitors has witnessed a significant advancement in the preclinical and clinical characteristics of drugs. Their classification into different generations, with second generation drugs being the most recently explored in trials and introduced in the clinical practice, reflects their improved selectivity on the primary target (BTK), reduced inhibition of additional targets with mitigation of off‐target and off‐tissue adverse drug reactions and better therapeutic index (this one being the ratio between toxic and therapeutic active drug concentrations). The improvement in the therapeutic index allows better patient management owing to the reduction of the adverse events.

In addition to this, molecular dynamic studies have shown that covalent BTK inhibitors are type II kinase inhibitors and the binding affinity, as measured by equilibrium dissociation constant, is very high for all drugs, due to their covalent binding to cysteine 481. In addition to this, the inactivation rate is very fast, with k_inact_ (s^−1^) of acalabrutinib of 5.59 × 10^−3^ followed by ibrutinib (2.66 × 10^−2^) and zanubrutinib (3.33 × 10^−2^)^5^ The substitution of the cysteine residue with a different amino acid, that is, serine, strongly increases the dissociation constant, due to the lack of the substrate for covalent reaction and is a major determinant of resistance to covalent BTK inhibitors.

On clinical grounds, better results have been achieved with newer (i.e., later‐generation) agents, with zanubrutinib showing more favourable results than ibrutinib and acalabrutinib being at least as active as ibrutinib, according to the available results of randomized trials performed in several disease contexts.^18^, ^24^, ^25^, ^38^, ^39^ Comparisons across studies with different agents, albeit with the limitations of this operation, have more in general showed a tendency to better efficacy of newer versus older agents (Table 1): it should be noted, however, that more heavily pretreated and poorer‐risk patients have been enrolled in earlier than in more recent trials. Besides that, we have learned that BTK inhibitors work better when applied early in the course of any disease.

**TABLE 1:** Efficacy of BTK inhibitors in lymphoid B‐cell malignancies. Indirect comparisons are made across studies with different inhibitors in each malignancy when no randomized studies have been performed. In case of available studies implying a head‐to‐head comparison between two BTK inhibitors, results from each arm are reported. Data on zanubrutinib are reported in bold and italics.

		Pts	ORR	CR	VGPR	PR	SD	PD	TTR	PFS	OS	References
TN‐CLL (^a^)	Ibrutinib	136	92.0	30.0	—	62.0	4.0	NR	NR	59.0 at 84 mos	78.0 at 84 mos	13, 15
	Acalabrutinib	179	92.2	11.2	—	81.0	2.2	1.7	NR	77.9 at 48 mos	87.6 at 48 mos	^19^
	***Zanubrutinib***	***241***	***94.5***	***6.6***	***—***	***87.9***	***2.9***	***0.8***	***NR***	***85.5 at 24 mos***	***94.3 at 24 mos***	^25^
RR‐CLL (^a^)	Ibrutinib	195	90.0	8.0	***—***	82.0	NR	NR	NR	40.0% at 60 mos	Median NR	12, 14
	Ibrutinib	265	79.9	3.0	—	76.9	10.2	2.3	NR	Median 38.4 mos	Median NR	^18^
	Acalabrutinib	268	83.2	1.9	—	81.3	10.8	0.7	NR	Median 38.4 mos	Median NR	
	***Zanubrutinib***	***327***	***90.6***	***4.0***	—	***86.6***	***6.1***	***0.9***	***NR***	***78.4 at 24 mos***	***Median NR***	^24^
	Ibrutinib	325	82.8	2.5	—	80.3	10.8	2.2	NR	65.9 at 24 mos	Median NR	
Mantle cell	Ibrutinib	111	67.0	23.0	—	44	NR	NR	1.9	31.0 at 24 mos	47 at 24 mos	26, 27
	Acalabrutinib	124	81.0	40.0	—	41.0	9.0	8.0	1.9	49.0 at 24 mos	72.4 at 24 mos	28, 29
	***Zanubrutinib***	***112***	***84.8***	***NR***	***—***	***NR***	***NR***	***NR***	***NR***	***51.4 at 24 mos***	***75.9 at 24 mos***	^30^
Waldenström	***Zanubrutinib***	***102***	***81.4***	***0***	***36.3***	***45.1***	***2.9***	***1.0***	***2.83***	***78.3 at 42 mos***	***87.5 at 42 mos***	38, 39
	Ibrutinib	99	79.8	0	25.3	54.5	3.0	2.0	2.92	69.7 at 42 mos	85.2 at 42 mos	
	Acalabrutinib	106	93.0	0	7.8	72.9	NR	NR	4.6	82.0 at 24 mos	89.0 at 24 mos	^36^
Marginal zone	Ibrutinib	60	58.0	10.0	—	48.0	30.0	5.0	5.6	32.0 at 33 mos	72.0 at 33 mos	^40^
	Acalabrutinib	40	52.5	12.5	—	40.0	47.5	0	3.0	67.0 at 12 mos	91.4 at 12 mos	^41^
	***Zanubrutinib***	***66***	***68.2***	***25.8***	***—***	***42.4***	***19.7***	***9.1***	***2.8***	***70.9 at 24 mos***	***85.9 at 24 mos***	^42^

*Note*: VGPR, rate of very good partial responses (for Waldenström macroglobulinaemia only). PR rates in patients with CLL are calculated differently among studies: in this table, PR is considered as the sum of partial responses + nodular partial responses + partial responses with persistent lymphocytosis.

Abbreviations: CLL, chronic lymphocytic leukaemia; CR, percentage of complete responses; NR, not reached; ORR, overall response rate (in percentage); OS, overall survival (expressed as percentage); PD, percentage of patients with progressive disease; PFS, progression‐free survival (expressed as percentage); PR, percentage of partial responses; Pts, patients; RR, relapsed/refractory; SD, percentage of patients with disease stability; TN, treatment‐naïve; TTR, time‐to‐response (any response or best response, if available), in months.

^a^ Data are expressed for entire patient populations regardless cytogenetic or molecular risk profile (see text for detailed breakdown).

The improvement in terms of the toxicity profile has been significant across generations (Table 2). Given that each molecule has a peculiar toxicity fingerprint, it is of paramount importance to consider patients' clinical characteristics, comorbidities and concomitant medications in order to operate wisely the best treatment choice.

**TABLE 2:** Incidence (percentage) of adverse events of higher severity and adverse events of particular interest in trials with zanubrutinib in non‐Hodgkin lymphomas and CLL. Comparisons with ibrutinib (shaded columns) are reported in the table only if a randomized trial was available in the appropriate context.

	Relapsed MZL	Relapsed MCL	Treatment‐naïve or relapsed Waldenström macroglobulinaemia		Treatment‐naïve CLL	Relapsed CLL	
			Zanubrutinib	Ibrutinib		Zanubrutinib	Ibrutinib
Any grade ≥3	48.5	53.6	74.3	72.4	53.0	67.3	70.4
AEs leading to dose reduction	0	4.5	15.8	26.5	NR	12.3	17.0
AEs leading to dose interruption	36.8	11.6	62.4	63.3	NR	50.0	56.8
AEs leading to discontinuation	7.4	12.5	8.9	20.4	8.3	15.4	22.2
AEs leading to death	7.4	8.0	3.0	5.1	4.6	10.2	11.1
Major haemorrhage	1.5	5.0	8.9	10.2	5.0	3.7	4.3
Infections, any grade	55.9	NR	79.2	79.6	62.1	71.3	73.1
Infections, grade ≥3	22.1	NR	21.8	27.6	16.3	26.5	28.1
Hypertension, any grade	4.4	12.0	14.9	25.5	14.2	23.5	22.8
Hypertension, grade ≥3	2.9	3.0	9.9	20.4	6.3	15.1	13.6
Atrial fibrillation, any grade	2.9	3.0	7.9	23.5	3.3	5.2	13.3
Atrial fibrillation, grade ≥3	1.5	2.0	2.0	8.2	0.4	2.5	4.0
Ventricular arrhythmia, any grade	1.5	NR	NR	NR	0.4	NR	NR
Ventricular arrhythmia, grade ≥3	0	NR	NR	NR	0.4	0	0.6

Abbreviations: AE, adverse event; CLL, chronic lymphocytic leukaemia; MCL, mantle cell lymphoma; MZL, marginal zone lymphoma; NR, not reported.

## AUTHOR CONTRIBUTIONS

**Alessandro Broccoli:** Conceptualization (equal); writing – original draft (equal); writing – review and editing (equal). **Marzia Del Re:** Conceptualization (equal); writing – original draft (equal); writing – review and editing (equal). **Romano Danesi:** Conceptualization (equal); writing – original draft (equal); writing – review and editing (equal). **Pier Luigi Zinzani:** Conceptualization (equal); writing – original draft (equal); writing – review and editing (equal).

## CONFLICT OF INTEREST STATEMENT

Alessandro Broccoli: Sandoz, Merck, Janssen, Menarini/Stemline (research support), Takeda (consultancy), Sandoz, Gilead, Takeda, Kyowa Kirin, GlaxoSmithKline, Beigene (advisory relationship), Astra Zeneca, Roche (honoraria). Marzia Del Re: Astellas, Astra Zeneca, Celgene, Novartis, Pfizer, Bio‐Rad, Janssen, Sanofi‐Aventis, Roche, Lilly, MSD and Ipsen (consultancy, advisory relationship, honoraria). Romano Danesi: GlaxoSmithKline, Roche, Novartis, Pfizer, Sanofi Genzyme, Astra Zeneca, Janssen, Serb, Lilly, Clovis, Gilead, and EUSA Pharma (consultancy, advisory relationship, honoraria). Pier Luigi Zinzani: MSD, EUSA Pharma, Novartis (consultancy), Secura Bio, Celltrion, Gilead, Janssen, Bristol Meyers Squibb, Servier, Sandoz, MSD, Takeda, Roche, Kyowa Kirin, Novartis, ADC Therapeutics, Incyte, Beigene (advisory relationship, honoraria).

## Data Availability

Data sharing not applicable to this article as no datasets were generated or analysed during the current study.
